# Improved discriminability of spatiotemporal neural patterns in rat motor cortical areas as directional choice learning progresses

**DOI:** 10.3389/fnsys.2015.00028

**Published:** 2015-03-06

**Authors:** Hongwei Mao, Yuan Yuan, Jennie Si

**Affiliations:** ^1^Electrical Engineering, School of Electrical, Computer and Energy Engineering, Arizona State UniversityTempe, AZ, USA; ^2^Graduate Faculty of the School of Biological and Health Systems Engineering, Arizona State UniversityTempe, AZ, USA; ^3^Affiliate Faculty of the Interdisciplinary Graduate Program in Neuroscience, Arizona State UniversityTempe, AZ, USA

**Keywords:** associative learning, action selection, agranular medial and lateral areas, plasticity, support vector machines

## Abstract

Animals learn to choose a proper action among alternatives to improve their odds of success in food foraging and other activities critical for survival. Through trial-and-error, they learn correct associations between their choices and external stimuli. While a neural network that underlies such learning process has been identified at a high level, it is still unclear how individual neurons and a neural ensemble adapt as learning progresses. In this study, we monitored the activity of single units in the rat medial and lateral agranular (AGm and AGl, respectively) areas as rats learned to make a left or right side lever press in response to a left or right side light cue. We noticed that rat movement parameters during the performance of the directional choice task quickly became stereotyped during the first 2–3 days or sessions. But learning the directional choice problem took weeks to occur. Accompanying rats' behavioral performance adaptation, we observed neural modulation by directional choice in recorded single units. Our analysis shows that ensemble mean firing rates in the cue-on period did not change significantly as learning progressed, and the ensemble mean rate difference between left and right side choices did not show a clear trend of change either. However, the spatiotemporal firing patterns of the neural ensemble exhibited improved discriminability between the two directional choices through learning. These results suggest a spatiotemporal neural coding scheme in a motor cortical neural ensemble that may be responsible for and contributing to learning the directional choice task.

## Introduction

When selecting an action among alternatives in response to an external stimulus, an animal usually makes its choice according to consequences of the actions taken. Animals choose those actions that have resulted in rewards in the past and thus, learning takes place by correctly associating a stimulus with an appropriate response. A neural network that underlies the acquisition of this stimulus-response association has largely been identified (Murray et al., 2000), and it points to the prefrontal cortex (PFC) and the basal ganglia as two key nodes for solving an associative learning task (Pasupathy and Miller, 2005). Within the frontal lobe, a rostro-caudal hierarchical organization supporting cognitive control functions such as action selection has been hypothesized (for a review see Badre, 2008). Primate studies have shown that premotor regions are also involved in learning and holding stimulus-response representations under the influence of prefrontal regions through top-down control (Koechlin et al., 2003; Boettiger and D'Esposito, 2005; Fluet et al., 2010). Additionally, the primary motor cortex has been suggested for encoding information beyond movement kinematics (Carpenter et al., 1999; Matsuzaka et al., 2007) such as features of visual stimuli that are behaviorally relevant (Zach et al., 2008; Eisenberg et al., 2011). Furthermore, the motor cortex is highly plastic for learning sensory-motor associations (Sanes and Donoghue, 2000). Putting it all together, the motor cortical regions in the frontal cortex are implicated for learning which action to select according to stimulus-response association.

Two types of adaptation could co-exist during sensorimotor association learning: motor skill learning that improves the execution of motor responses and associative learning that links sensory cues with specific response actions (Cohen and Nicolelis, 2004). Motor skill learning alone could induce neural plasticity ranging from synaptic connections (Xu et al., 2009), changing neural firing rates (Li et al., 2001; Kargo and Nitz, 2004; Rokni et al., 2007), to the motor map (Kleim et al., 1998, 2004) in both young and adult motor cortices. Therefore, motor skill learning could become a confound factor when studying sensorimotor association learning and should be treated with care. Aside from well-studied motor skill learning, whether and how motor cortical activity would adapt during associative learning is still unclear and requires further investigation.

In a previous study (Cohen and Nicolelis, 2004), rats learned to associate directional movements in response to either a high or a low tone. Significant neuronal firing rate changes in the primary motor cortex were observed on the first day when an animal's movement skill improved, but not in the following 2 days when movement parameters were stable and associative learning dominated. In some other studies where animals learned to respond to external sensory cues with appropriate actions, learning-related neural dynamics were evident in motor cortical neural ensemble activity patterns (Laubach et al., 2000; Huber et al., 2012). Based on these results we hypothesize that neural adaptation induced by learning sensorimotor associations would be reflected in changes in spatiotemporal neural firing patterns in motor cortical areas. To test this hypothesis, we had rats learn to perform a directional choice task. The goal of the task was to make a left or right side lever press in response to a left or right side light cue, respectively. Single units were recorded from rat's medial agranular (AGm) and lateral agranular (AGl) areas. Spatiotemporal neural firing patterns were investigated using support vector machines. Improved discriminability in neural patterns was observed as learning progressed.

## Materials and methods

### Animal handling and surgery

All procedures were in accordance with guidelines of the National Institutes of Health and approved by the institutional Animal Care and Use Committee at Arizona State University. Rats (Long-Evans, male) arrived at the age of about 2 weeks weighing around 50 grams and were handled daily by experimenters to get accustomed to the environment. They started pre-training after reaching 200 grams to master the motor skill of lever pressing, which only involved pressing a single lever (no choice) in response to a light cue above the lever. The pre-training apparatus was similar to that used for recording to help familiarize rats with the recording environment. After achieving a behavioral accuracy of 90% or above for at least 3 consecutive days on the pre-training task, and once their weight reached 400 grams, rats were implanted with a chronic electrode array.

**Figure 1:**
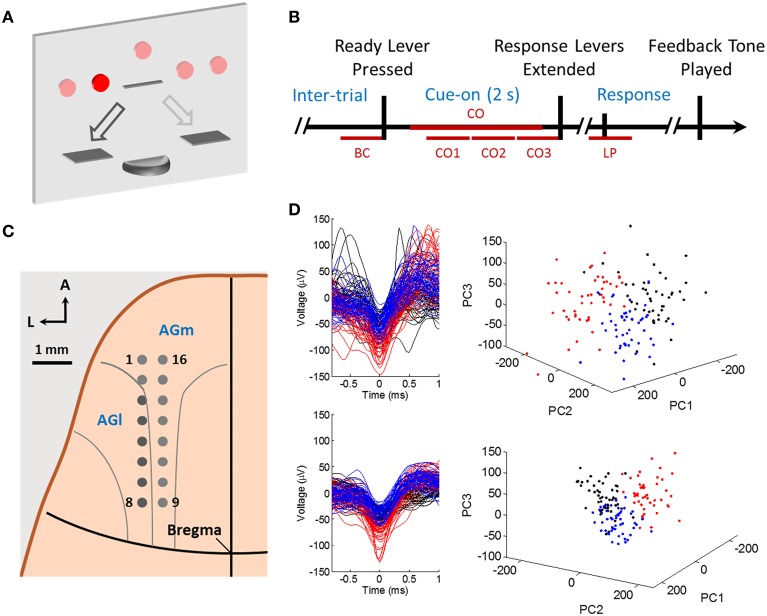
**The behavioral task, trial timeline, and recording sites. (A)** The control panel setup. The rat was cued by an LED light in one of five positions in any given trial, and could use the left and right response lever to “move” light to the right and left by one position, respectively. The goal was to reach the center light by appropriate lever presses in order to receive a sugar pellet reward. **(B)** The task timeline. The rat would self-start a trial by pressing the center ready lever at his own will, and simultaneously a light cue would turn on. Then there would be a 2 s cue-on period, after which the two response levers would extend simultaneously. The rat would choose and press one of the response levers. A feedback tone would be played at trial end indicating the outcome. **(C)** A 2 × 8 microwire array was chronically implanted in the left hemisphere of the rat brain, aiming for layer V AGm and AGl neurons. **(D)** Example single unit recordings from rat A09 (top row) and rat K11 (bottom row). Left: unsorted waveforms (black), spike waveforms used in neural analysis (blue) and waveforms of another unit not used due to very low firing rate (red). The 50 waveforms from each class were randomly selected and plotted. Right: 3-D PCA projection of the waveforms.

For electrode implant surgery, rats were anesthetized by an intramuscular injection of KXA (10 mg/ml ketamine, 2 mg/ml xylazine, and 0.1 mg/ml acepromazine; 0.1 ml/100g), shaved in the incision area, and placed in a stereotaxic frame. A heated water blanket was used to maintain rat's body temperature at around 35°C. Rat's heart rate and oxygen level were monitored throughout surgery with a pulse oximeter. KXA updates (0.05 ml/100g) were administrated approximately every hour during surgery after the initial shot. Craniotomy was performed over the AGm and AGl areas of the left hemisphere of the rat brain. A microwire array was centered at 2 mm lateral and 3 mm rostral from the bregma (Figure 1C), and lowered about 1.8–2.3 mm underneath dura, aiming for layer V pyramidal neurons. An acrylic head cap was formed to support the electrode array. The head cap was fixed to the skull with three screws. A subcutaneous injection of 0.1 ml meloxicam was given for pain relief after surgery, and three more shots were given for the following 3 consecutive days. The rats had 7–10 days or as needed to recover before they were food restricted for recording sessions.

### Behavioral task

Rats were freely moving in the recording chamber, and self-paced to start a trial by pressing the retractable center ready lever. One of the five cue lights (from left to right: LL, L, C, R, and RR) would appear (Figure 1A) immediately upon ready LP. The left and right response levers would extend 2 s after cue light onset (Figure 1B). Pressing the left lever once would “move” the light one position to the right and pressing the right lever would “move” the light to the left. Once the light cue reached and then remained at the center position for at least 1 s, the trial ended as a success. Otherwise a trial was considered a failure if the light cue ended up at any position other than the center. A feedback tone was played immediately upon the end of a trial: a low frequency tone of 1 kHz in case of a success and a high frequency tone of 12 kHz in case of a failure. A sugar pellet reward was delivered 0.5 s after the feedback tone for a successful trial. The inter-trial interval was 8 s for successful trials and 15 s for failed trials. The five cues were presented in a pseudo-random fashion with equal probability of presence.

### Recording sessions

After rats recovered from surgery, daily recording sessions began, each of which lasted about 60 min. Rats were food restricted during the recording period while the body mass was closely monitored.

The implanted electrodes were arranged in a 2 × 8 matrix, with 500 or 375 μm row separation, and 500 μm electrode spacing. The polyimide-isolated tungsten microwires were 50 μm in diameter and 5 mm in length. The electrode tips were cut at a sharp 60° angle (TDT Inc., FL). A total of 16 channels of raw waveforms were recorded simultaneously using a RX5 Pentusa Base Station or a RX7 Microstimulator Base Station (TDT Inc., FL). Neural signals picked up by electrodes were passed to a unity gain preamplifier (bandpass 2.2 Hz ~ 7.5 kHz) through an Omnetics or a ZIF-Clip headstage, and then sampled and stored at 24.414 kHz by the base station.

Rat behavior while performing the task was monitored and recorded using cameras (25 fps). Rat head position was determined offline by the implanted head cap as well as left and right ear positions. The head position was tracked and extracted to indicate rat movement trajectory, which was calculated for left side and right side movements separately over recorded trials. Variation in movement trajectory was obtained as the distance between a given movement trajectory during a trial and the mean trajectory in a single recording session.

### Spike sorting

Action potentials were detected and classified off-line using our own M-Sorter software (Yuan et al., 2012), which is based on the multiscale correlation of wavelet coefficients (MCWC) detection algorithm (Yang et al., 2011). The M-Sorter has been tested and compared with two popular sorters: the Wave Clus and the automatic mode of Offline Sorter by Plexon (T-Distribution EM method). The M-Sorter consistently outperformed or was at least comparable to the compared sorters (Yuan et al., 2012). One isolated unit with highest firing rate was extracted from each of the electrodes. Experimenters also inspected spike waveforms, inter spike intervals, and other measures to ensure the quality of single unit clusters (Figure 1D). According to the sites of implanted electrodes, recorded neurons were in the AGm and AGl areas of the rat frontal cortex (Paxinos and Watson, 2005) involving forelimb, neck, and vibrissae areas (Neafsey et al., 1986; Remple et al., 2001). Intracortical microstimulation was also performed to confirm implant electrode location.

For each rat, only those electrodes that consistently picked up unit action potentials in all sessions were included in neural activity analysis. By doing so, we were able to analyze neural ensembles of the same size over sessions to make the results comparable. The analyses in this study as described below were based on neural ensembles. Therefore, we did not require tracking same neurons over learning sessions (Laubach et al., 2000; Cohen and Nicolelis, 2004).

### Firing rate modulation

In this study, L-L trials are used to denote those trials in which rats reported left side choices by pressing the lever on the left side in response to left side cues, and similarly we define R-R trials. Single unit firing rates in single trials were calculated using a 100 ms data window sliding at 20 ms steps (50 bin/s) through the cue-on task period (Figure 1B). The mean firing rate for a data window (CO1, CO2, CO3, or CO) was the average of all binned firing rates in the respective data window.

Let the *i*th neuron's mean firing rate in session k for all L-L and R-R trials be denoted as *M^i^_L_*(*k*) and *M^i^_R_*(*k*), respectively. The *i*th neuron's mean firing rate was then defined as $M^{i}(k)=\frac{1}{2}(M_{L}^{i}(k)+M_{R}^{i}(k))$. The *i*th neuron's firing rate difference between L-L and R-R trials in session k was calculated as *D^i^*(*k*) = *M^i^_L_*(*k*) − *M^i^_R_*(*k*).

The ensemble mean firing rate *M*(*k*) of *N* isolated units in session *k* was the average over all recorded trials of isolated units, i.e., $M(k)=\frac{1}{N}\sum_{i\;=\;1}^{N}M^{i}(k)$.

The ensemble mean firing rate difference between L-L and R-R trials was then the average of the absolute value of single unit rate differences, i.e., $D(k)=\frac{1}{N}\sum_{i\;=\;1}^{N}|D^{i}(k)|$. As such, each recording session corresponded with one measurement for the ensemble mean rate and another measurement for the ensemble mean rate difference.

In addition to firing rates, firing variability was also monitored. First, we calculated the standard deviation of firing rate of unit *i* in session *k*, *S^i^_L_*(*k*) and *S^i^_R_*(*k*), for L-L and R-R trials, respectively. Then, the mean standard deviations, *S_L_*(*k*) and *S_R_*(*k*), were calculated as the average across units, respectively.

To study how the ensemble mean firing rate and ensemble mean rate difference would change during learning from session to session, linear regressions were performed against normalized session numbers (between 0 and 1). The sign of the regression line slope was determined according to its confidence interval. A positive slope corresponded with increased rate measures while a negative slope with decreased rate measures. No change in rate measures was associated with a regression line slope that was not significantly different from zero. Similar linear regression analysis was used to examine changes of other measurements as described below.

In order to summarize results of multiple rats, the ensemble mean firing rates of single sessions were Z-scored (zero mean and standard deviation equal to one) over sessions for each rat. Then Z-scored ensemble mean rates from all rats were pooled together for linear regression analysis. Other measurements, including ensemble mean rate difference, mean standard deviation of firing rate, and SVM classification results as described below, were processed in a similar manner when their trends over sessions were explored by summarizing multiple rats' data.

### SVM classification of neural representations

We modeled neural firing patterns of L-L and R-R trials by training linear kernel support vector machines (SVMs). The input to the SVMs was spatiotemporal neural firing activity in the cue-on task period of a single trial while the output of the SVMs was the directional choice of left or right. All analyses were performed using customized Matlab programs (Mathworks Inc., MA).

SVMs solve a binary classification problem by determining a separating hyperplane with a maximized margin between two classes (Burges, 1998). Once the separating hyperplane is found, an SVM makes a classification decision for a given data sample x according to the value of the decision function: *df* (***x***) = ∑_*i*_α_i_*K* (***s***_***i***_, ***x***) + *b*, where support vector ***s_i_***, weight α_*i*_ and bias *b* are determined in the training process automatically once input and output data are presented for training, and the kernel function K is a dot product in case of a linear kernel. If *df* (***x***) ≥ 0, ***x*** is classified as an L-L trial, otherwise it is classified as an R-R trial. The decision function value could be interpreted as the distance from the sample point to the separating hyperplane. The greater this distance the less ambiguous the final classification.

**Figure 2:**
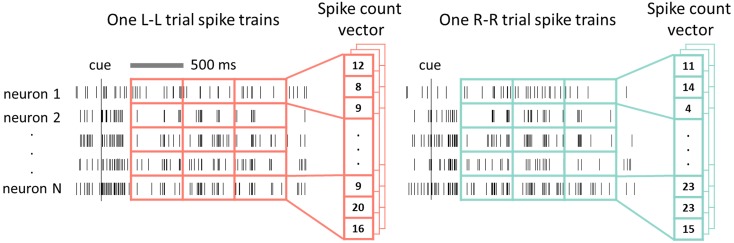
**Data preparation for SVM decoding**. Spike trains of all simultaneously recorded single neurons from one task trial formed one data sample where the spike counts in non-overlap bins (e.g., 500 ms bins) were concatenated to form a spike count vector. Two classes of data samples from L-L and R-R trials composed the data set.

In our analysis, a 1500 ms data window in the cue-on period (CO: 300 to 1800 ms after cue onset, Figure 1B) was used. This window was divided into three non-overlapping 500 ms time bins. Spike counts in these bins formed one vector representation for each spike train of each unit. Spike count vectors of simultaneously recorded units were then concatenated to form a spike count vector representation of the recorded neural ensemble (Figure 2). Thus, there was one ensemble vector or one data sample for each trial, and SVMs were trained based on data samples from both classes (L-L and R-R trials) in each recording session for each rat.

To obtain statistically representative results, a total of 100 SVM classifiers were trained and tested for each session. In each of the 100 classifiers, a constant number of trials were randomly chosen from both L-L and R-R classes. Specifically, 20% of the randomly chosen trials from each class formed the test set, and the remaining 80% formed the raw training set, which was further processed as follows before training an SVM. First, five trials of the same class were randomly selected from the raw training set, each with a respective spike count vector. Then an average spike count vector was obtained based on the five spike count vectors, and was used as an input training data sample for the SVM model. A test data sample simply was a single spike count vector of a test trial. The SVM performance measure was based on averaged test set classification accuracy from the 100 SVM classifiers. This procedure was repeated for each recording session over the entire directional choice learning process. SVM based classification performance of L-L vs. R-R trials over multiple sessions were then inspected using linear regression.

As a control, SVM classification analysis was also performed using a 500 ms time window around response lever press (LP, −100 to 400 ms around the press, Figure 1B). Data was prepared in a similar way as described above and 100 ms time bins were used to compute spike counts. To make classification performance comparable, three 500 ms windows within the cue-on period were selected (CO1, 500 to 1000 ms after cue onset; CO2, 1000 to 1500 ms; CO3, 1500 to 2000 ms; Figure 1B). SVM classification analysis was repeated in these data windows and compared with that using data in LP.

Note that in our analysis, 100 ms bin size was used for 500 ms data window (CO1, CO2, CO3, and LP) based direction predictions by SVM. But for SVM classification analyses where the CO window (Figure 2) was involved, 500 ms bins were used to form spike count vectors.

## Results

### Behavioral results

**Figure 3:**
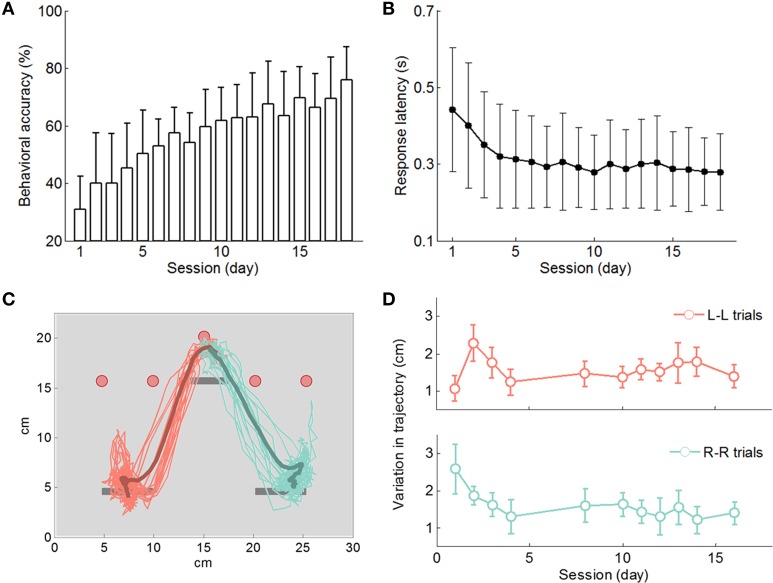
**Behavioral results. (A)** Rats (*n* = 9) improved behavioral accuracy to a level above 70% in 18 days on average. **(B)** The response latency decreased mainly during the initial days and stayed relatively stable afterwards. **(C)** Movement trajectory (head position) during the cue onset period projected onto the front view of the interface panel. Thin traces: trajectory records from single trials. Bold curves: mean trajectory traces of left side and right side movements. **(D)** Variation in movement trajectory (distance between the trajectory trace of a single trial and the mean trajectory) decreased during initial learning sessions.

Male Long-Evans rats (*n* = 9) started learning the directional choice task by trial and error from a naïve state. Behavioral accuracy in each recording session was monitored and calculated as the number of correct trials over the total number of trials in that session. Rats gradually improved the accuracy over sessions, from 30.8% (average, range from 14.1 to 47.3%) in session 1 to 76.0% (average, range from 55.3 to 93.4%) in session 18 (rank-sum test, *p* < 0.001; Figure 3A). Linear regressions of behavioral accuracies vs. session numbers revealed that seven rats significantly improved their performance except rat A09 and I10. Actually rat A09 didn't learn the right side choices, and rat I10 struggled with both left and right side choices. Among the seven rats, one of them (J11) reached 75% accuracy, all the other six rats went above 80%, and two rats (W09 and O10) even achieved over 90% accuracy. We therefore used data from the seven rats when reporting results against normalized session numbers as learning progressed. When results are based on data from all nine rats, it will be specified accordingly.

Once the rat self-started a new trial (Figure 1B), he had 2 s to choose from the two response levers prior to their extension. Upon response lever extension, he could make a press of his choice within 1 s. In this analysis, the response latency was calculated as the time from response lever extension to the first press on the chosen lever. This latency decreased from 0.44 ± 0.16 s in session 1 to 0.32 ± 0.14 s in session 4 (mean ± STD; ANOVA, *p* < 10^−5^; Figure 3B; nine rats). After the first three learning sessions the response latency became stabilized, and from the fourth session onwards, the latency measurements over sessions were not significantly different (linear regression slope at −0.0021 s/session, 99% confidence interval of [−0.0058 0.0016]). The reduction in response latency is an indicator of improved act of lever pressing.

During task performance, rats usually made quick movements toward their chosen lever right after light cue onset and their movements became stereotypical in a few sessions. Video analysis of rat movement trajectory during the cue-on period (Figure 3C) confirmed this observation. Typically, rats started moving in their chosen direction shortly after cue onset. Their directional movements were completed by about 1 s after cue onset and then rats stayed in front of the response lever waiting for lever extension. To measure differences in rat directional movement from trial to trial, variation in movement trajectory between a single-trial trajectory and the mean trajectory of a session was calculated for each session. As shown in Figure 3D, variations in movement trajectory decreased during the first three sessions and remained stable afterwards. Additional video analysis results of rats' directional movement trajectory are available in Yuan et al. (2014). These observations together with response latency results show that motor skill learning occurred during the first few days and therefore, it could be dissociated from the rest of the associative learning process.

### Firing rate modulation by directional choice

In this study, trials with correct first response lever press were used in analysis and within those trials, we mainly focused on the cue-on task period (Figure 1B). A total of 220 sessions were recorded from nine rats. We included 190 sessions for analysis and excluded the remaining 30 sessions because of inadequate numbers of trials (less than 20 L-L or 20 R-R trials). Those sessions mainly included the first few sessions of each rat when behavioral accuracy was low and motor skill learning was possibly present. All together for this study, we had 11,060 L-L trials and 10,717 R-R trials from the 190 sessions with 58 L-L trials and 56 R-R trials per session on average. Of the 190 recorded sessions from nine rats, we collected 839 unit records (337 from AGl and 502 from AGm), 4.4 unit records per session on average, ranging from 3 to 6 unit records in the ensemble. Here we consider an isolated unit each day a unit record.

**Figure 4:**
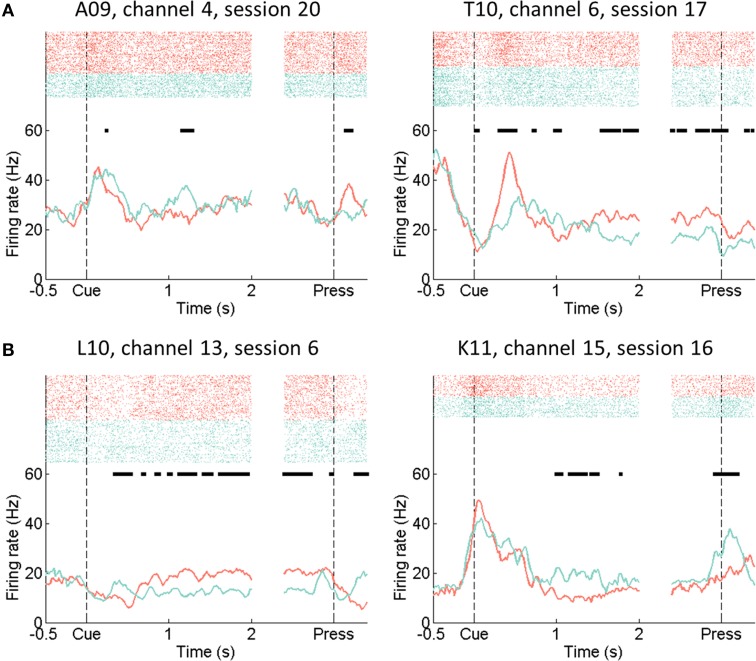
**Raster plots and peri-event time histograms of example single units**. Firing rate estimated in 100 ms time bins sliding down the time axis with a step size of 20 ms. Red: L-L trials in which the rat moved to the left response lever in response to left side cues. Green: R-R trials with right side movements in response to right side cues. **(A)** Example AGl units. **(B)** Example AGm units. Horizontal bars (black) indicate time bins in which directional firing rate modulation was significant (rank-sum test, *p* < 0.01).

Single unit firing activities of L-L and R-R trials in each session were first inspected by spike rasters and peri-event time histograms (PETHs). Examples of single unit firing rate modulations by L-L and R-R trials are shown in Figure 4.

**Figure 5:**
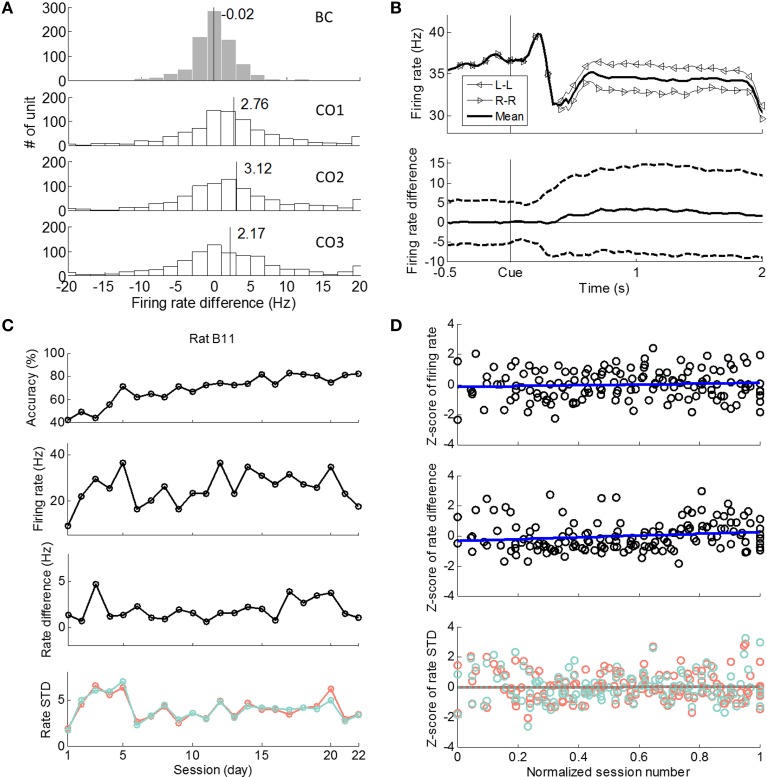
**Firing rates and firing rate differences between L-L and R-R trials. (A)** Histograms of single unit (*n* = 839) firing rate differences between L-L and R-R trials for nine rats in four 500 ms time windows. BC: 500 ms window before cue onset. CO1: 500 to 1000 ms after cue. CO2: 1000 to 1500 ms after cue. CO3: 1500 to 2000 ms after cue. **(B)** Top: averaged (over all units, nine rats, and sessions) single unit firing rates, *M^i^_L_*(*k*) for L-L trials, *M^i^_R_*(*k*) for R-R trials, and *M^i^*(*k*) for mean firing rate over L-L and R-R trials. Bottom: mean ± STD of single unit firing rate difference, *D^i^*(*k*). **(C)** Rat B11 as an example. Ensemble mean firing rate and rate difference between L-L and R-R trials in CO data window (300 to 1800 ms after cue onset). Four panels from top to bottom: (1) behavioral accuracy; (2) ensemble mean firing rate over sessions; (3) ensemble mean rate difference between L-L and R-R trials over sessions; (4) averaged standard deviation of firing rate over sessions for L-L (red) and R-R (green) trials. **(D)** Linear regression of Z-scored ensemble mean firing rate (top) and ensemble mean firing rate difference between L-L and R-R trials (middle) of seven rats in the CO data window. Linear regression of averaged standard deviation (in Z-score) for L-L (red) and R-R (green) trials of seven rats in the CO data window (bottom).

In the 500 ms time window before cue onset, the averaged (over all single units, nine rats, and sessions) single unit firing rate difference between L-L and R-R trials was −0.02 Hz, which was not significantly different from zero (one-sample *t*-test, *p* > 0.89). If the same single unit firing rate differences between L-L and R-R trials were evaluated in three cue-on sub-windows (CO1, CO2, and CO3 in Figure 1B), they were 2.76, 3.12, and 2.17 Hz, respectively, all of which were significantly greater than zero (one-sample *t*-test, *p* < 10^−5^; Figure 5A).

We then evaluated time-resolved (100 ms bins for every 20 ms) single unit firing rates of L-L and R-R trials, and the firing rate difference between the two during the cue-on period (Figure 5B). For the pool of single units, the averaged (over all single units, nine rats, and sessions) time-resolved firing rate difference did not emerge from 0 until 400 ms after cue onset (one-sample *t*-test, *p* < 0.001), and it sustained through the rest of the cue-on window. These results show that firing rate modulation of single neurons was prominent in motor cortical areas during the cue-on period.

To study how firing rate modulation at a population level varied as learning progressed, we calculated the ensemble mean rate and ensemble mean rate difference between L-L and R-R trials (see Materials and Methods) session by session. The results from using rat B11's data are given in Figure 5C as an example where the firing rates of a 1500 ms cue-on window (CO, Figure 1B) were used. To summarize results from all seven rats, we normalized session numbers. According to Figure 5D, the Z-scored ensemble mean firing rate did not change significantly through the learning process (99% confidence interval of linear regression slope: [–0.48 1.05]). When the learning process was divided into three stages of equal numbers of sessions, the Z-scored ensemble mean rates were −0.21 ± 1.08, 0.16 ± 0.85, and 0.00 ± 1.00 Hz (mean ± STD), which were not significantly different (ANOVA, *p* > 0.1). The Z-scored ensemble mean rate difference between L-L and R-R trials tended to increase slightly with a slope of 0.59 (Figure 5D), but its 99% confidence interval was [−0.16 1.34] indicating it was not significantly different from zero. When calculated in the three learning stages, the Z-scored ensemble mean rate differences were −0.16 ± 1.09, −0.19 ± 0.83, and 0.32 ± 0.96 Hz, respectively, showing higher rate differences between L-L and R-R trials in the last stage compared with the previous two stages (ANOVA, *p* < 0.05). But Figure 5D also shows that some early session had large rate differences. When linear regression was performed for individual rats, the slope was again not significantly different from zero (*t*-test, *p* > 0.15). To summarize, although ensemble mean rate difference tended to become larger near the end of the recorded learning process, the trend was not strongly observed.

The BC data window (Figure 1B) was analyzed in a similar way to provide a control. The ensemble mean firing rate leveled over sessions (slope of Z-scored rates: 0.04, 99% confidence interval of slope: [−0.73 0.81]; Z-scored rates in the three stages were: −0.16 ± 1.06, 0.21 ± 0.77, −0.09 ± 1.08, ANOVA *p* > 0.1). The ensemble mean rate difference was relatively stable over sessions as well (slope of Z-scored rate difference: −0.40, 99% interval: [−1.17 0.36]; Z-scored rate difference in the three stages were: 0.17 ± 1.01, −0.04 ± 0.92, and −0.09 ± 1.01, ANOVA *p* > 0.4).

Additionally, the standard deviation of firing rate during the CO data window remained relatively stable across learning sessions. The linear regression slope of mean standard deviation for L-L trials against normalized session number was 0.06, which was not significantly different from zero given that the 99% confidence interval of the slope was [−0.70 0.82] (Figure 5D, red dotted line). Similarly for R-R trials, the linear regression slope was 0.04 and its 99% confidence interval was [−0.72 0.80] (Figure 5D, green dotted line).

To summarize, the ensemble mean firing rate of all simultaneously recorded single units over all trials did not change significantly over sessions, and the ensemble mean firing rate difference between L-L and R-R trials did not show a clear trend of change either. These findings suggest that ensemble mean rate based measures are not adequate to explain improved behavioral learning of the seven rats under study.

### Spatiotemporal firing pattern analysis by SVMs

As learning related behavioral adaptation could not be well-explained by ensemble mean firing rate or ensemble rate difference between L-L and R-R trials in cue-on period, we then inspected the neural data with increased spatiotemporal resolution using SVMs. In the following, we first examined how data preparation and SVM parameters may affect SVM model performance.

#### Ensemble vs. Single Units

**Figure 6:**
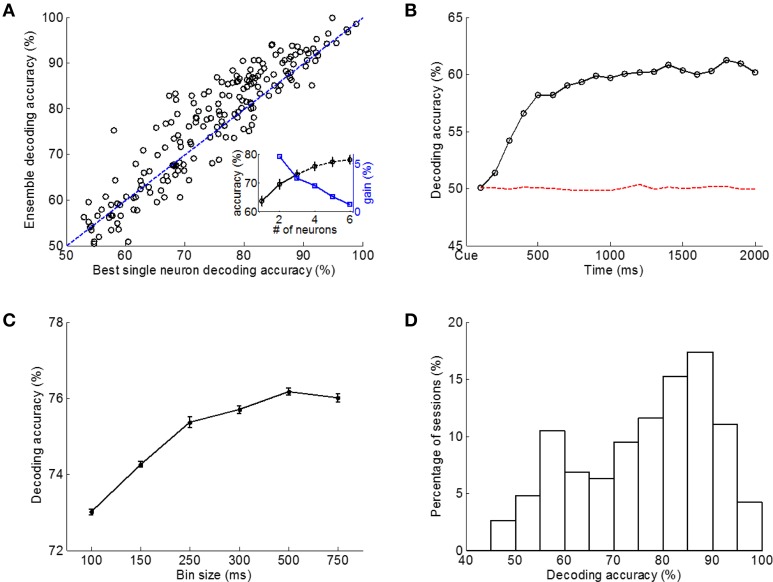
**Decoding accuracy between L-L and R-R trials using linear SVM classifiers with different data and classifier configurations. (A)** Classification performance using neural ensembles vs. best single neurons. CO data window, 500 ms time bins. Each point represents one of the 190 sessions, and those above the diagonal line indicate better classification performance using the ensemble. Insert: decoding accuracy increased when larger ensembles were used, but the speed of increase in decoding accuracy by using larger ensembles slowed down as ensemble size increased. **(B)** SVM classification accuracy using data of single 100 ms time bins, averaged over all sessions with chance level classification accuracy (dashed line) obtained using shuffled training data. **(C)** Bin size affected classification performance. **(D)** Histogram of single session SVM classification performance (190 sessions). Averaged classification accuracy (test data set) was 76.18% based on CO data window with 500 ms bins.

Ensemble spike count vectors were formed by concatenating spike counts (CO window, 500 ms bins) of simultaneously recorded single units. For each session, SVMs were trained and tested using ensemble vectors [vector dimension was 3× (number of single neurons)], and classification performance was characterized by classification accuracy on the test data. On the other hand, SVMs were built and tested using spike count vectors of single units (three dimensional vectors). When comparing the best classification performance using single unit data with that using ensemble data (Figure 6A), the ensemble approach outperformed the best single unit approach in 61.6% (117/190) of tested sessions. The mean single trial decoding accuracy among all sessions when using ensemble approach was 76.2%, which was higher than the 74.2% accuracy of the best single units (paired-sample *t*-test, *p* < 10^−5^).

Additionally, we examined the impact of the ensemble size on decoding accuracy. Each of the nine rats had at least 3 units per session. Specifically, one rat had 3 units per session, four rats had 4 units per session, three rats had 5 units per session, and one rat had 6 units per session. As shown in Figure 6A insert, decoding accuracy increased when larger ensembles were used, but the speed of increase in decoding accuracy by using larger ensembles slowed down as ensemble size increased.

#### Multiple vs. Single Time Bins

To explore SVM classification performance over time, the cue-on period was divided into non-overlapping 100 ms time bins and SVMs were trained using data of spike counts in a single time bin from all simultaneously recorded units. As shown in Figure 6B, the classification accuracy (averaged over all sessions) gradually increased after cue onset and then leveled off at around 60% (still above chance level of 50.02% accuracy when training samples from both classes were randomly shuffled, one-sample *t*-test, *p* < 10^−5^). This is consistent with our previous observation of sustained firing rate modulation between L-L and R-R trials during cue-on period (Figure 5B). As a comparison, when spike counts in 15 bins (100 ms bin width) together (CO window) were used in SVM model for classification, the average decoding accuracy was 73.01% over all sessions, which was significantly higher than the decoding accuracy when single time bins were used (paired-sample *t*-test, *p* < 10^−5^). Therefore, temporal firing patterns or spike counts in multiple consecutive time bins were expected to benefit SVM neural decoding.

#### Size of Time Bin

Then we tested how the size of a time bin may affect SVM classification. The same 1500 ms (CO window) neural ensemble data was used but spikes were counted in non-overlap time bins of different sizes, ranging from 100 to 750ms. Best classification performance was obtained using 500 ms bins with a 76.18% decoding accuracy (Figure 6C). Larger time bins (e.g., 750 ms) resulted in slightly lower classification accuracy (76.01% accuracy; paired-sample *t*-test, *p* < 0.01) probably due to loss of temporal resolution. However, higher temporal resolutions did not help improve classification accuracy either (paired-sample *t*-test, *p* < 0.01). Given the above discussion, we used spike counts in 500 ms bins for analyses hereafter unless otherwise specified.

The histogram of classification performance in single sessions is shown in Figure 6D, where spike counts in 500 ms non-overlapping bins during the 1500 ms CO window of neural ensembles were used for decoding. The mean classification accuracy tested with novel single trial data was 76.18% for all sessions from all rats.

To gain additional insight into the firing patterns during the CO window, SVM decoding analysis was also performed between correct and error trials. Two classes of error trials were considered here: R-L trials stand for those left side choices in response to right side cues, and similarly we define L-R trials. Only 11 out of all 190 sessions had at least 20 trials per class for L-L vs. R-L analysis, and 18 sessions were included in R-R vs. L-R analysis. The average decoding accuracy for L-L vs. R-L trials across the 11 sessions was 53.74% which was slightly but significantly higher than chance (t-test, *p* < 0.03), and the accuracy for R-R vs. L-R decoding was 50.79% which was not significantly different from chance (*t*-test, *p* > 0.60). Specifically in L-L vs. R-L analysis, decoding accuracy was higher than chance in 7 out of the 11 sessions (mean accuracy was 57.05%), lower than chance in one session (44.86% accuracy), and not different from chance in the remaining three sessions (*t*-test, α = 0.001). For the 18 sessions included for R-R vs. L-R analysis, these numbers were six sessions (58.58% accuracy), six sessions (44.38% accuracy), and six sessions. Alongside these decoding results, it's also worth noticing rat behavioral accuracy data. The average behavioral accuracy was 69.1% for the 11 sessions used for L-L vs. R-L decoding, and the behavioral accuracy for the 18 sessions used for R-R vs. L-R decoding was 59.9%. To summarize, when the same directional choice was made in both correct and error trials (e.g., left side choices in both L-L and R-L trials), the neural patterns associated with the two types of trials were largely similar, but there still seemed to be some difference between the two. When the rat was less clear about correct vs. wrong choices (low behavioral accuracy), the neural activities were more similar for correct (R-R) and wrong (L-R) trials. However, this analysis is not conclusive due to limited data available (only 28 out of all 190 sessions were eligible for this analysis).

### Adaptation of spatiotemporal firing patterns with learning

**Figure 7:**
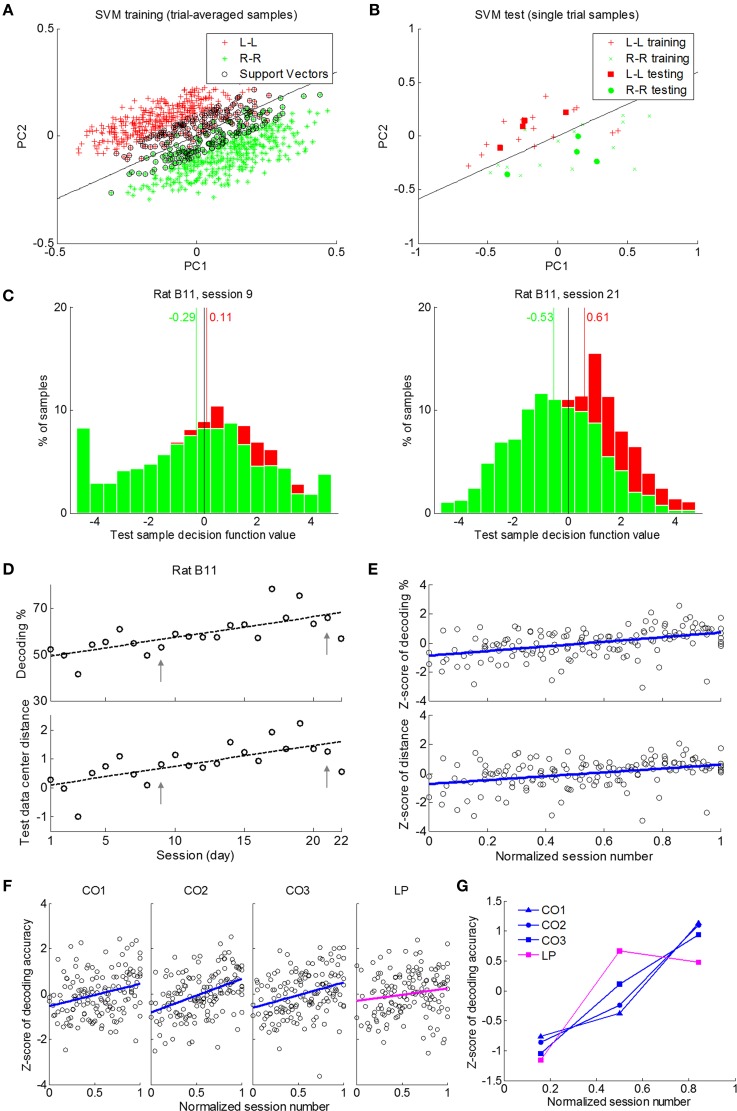
**SVM decoding over learning sessions**. The CO data window with spike counts in 500 ms time bins was used in all results except those in **(F)** and **(G)** where 100 ms bins were used. **(A)** An illustration of training data samples, support vectors and separating plane (black line) in a 2-D PCA projected space. Data of rat B11 in session 21 were used as an example. **(B)** The original single trial data samples used to generate trial-averaged training data and single-trial test data samples plotted in the same space as in **(A)**. **(C)** Histograms of decision function values of test data set for sessions 9 and 21 of rat B11. **(D)** An example of classification performance over sessions using data of rat B11. Upper panel: Classification accuracy tended to increase with learning. Lower panel: the distance between L-L and R-R data sets in the SVM kernel space increased with learning. **(E)** Classification accuracy and distance between the two classes in SVM kernel space increased through the course of learning (seven rats). **(F)** The increased decoding accuracy was significant in the three cue-on period data windows (CO1, CO2, and CO3), but not in LP window around response lever press. **(G)** Neural activity patterns of response lever press (LP) showed different dynamics in terms of classification accuracies (Z-scored) in three stages of the learning process compared with those in cue-on data windows (CO1, CO2, and CO3).

Before presenting evidence on neural adaptation as learning took place, we first illustrate how SVMs can be used for this purpose. Figure 7A is an example of how SVM classification took place to separate L-L and R-R trials where in the figure, we showed the first two principal components of the original spatiotemporal neural ensemble data. As shown, training data samples of the two classes formed distinct clusters and the SVM created an optimal separating line properly. This classifier was then used to predict rat response of left or right side lever press given a novel single trial neural data sample from the test data set (Figure 7B). The classification accuracy on the test set and the averaged decision function values (see Materials and Methods) of test samples from both classes could be calculated.

Figure 7C shows histograms of decision function values of test neural data samples from the 100 runs of randomly selected test samples in two sessions (session 9 and 21) from rat B11 as an example. Decision function values from the two sessions were significantly different between the two classes (ANOVA, *p* < 10^−5^). But the distance between the mean decision function values of the two classes (0.40 vs. 1.14) was larger in the later session when SVM classification accuracy was also higher (53.08% vs. 64.30%).

As classification accuracy increased over learning sessions, we also see an increase in the distance measurements of decision function values of the two classes provided by SVM (Figure 7D, rat B11 as an example). Among the seven rats, linear regression of Z-scored classification accuracy (CO data window, 500 ms bins) against normalized session number had a positive slope of 1.59 (Figure 7E), with the 99% confidence interval at [0.91 2.26]. If we divide learning sessions into three stages of equal length, the average of the Z-scored classification accuracy gradually increased over the three stages at −0.52, −0.19, and 0.48 (1st vs. 3rd stage: ANOVA, *p* < 0.001; Figure 7E). The Z-scored distance measurement between decision function values of the two classes had a positive regression slope of 1.32 (99% confidence interval at [0.62 2.02]), and increased significantly over the three stages as well to reach their respective Z-scored distance measurement of −0.44, −0.15, and 0.40 (1st vs. 3rd stage: ANOVA, *p* < 0.001; Figure 7E). These results suggest enhanced discriminability in spatiotemporal neural activity patterns between L-L and R-R trials as learning progressed.

After examining neural activities in the cue-on period in relation to rat's behavioral learning improvement, we attempted to gain additional insight by investigating neural activity patterns during the response lever press period as a control. A 500 ms time window (LP window, from −100 to 400 ms) around response lever press was used, and spike counts in 100 ms non-overlapping bins were used to build SVMs to decode L-L and R-R trial lever presses. For this time window, Z-scored classification accuracy exhibited a weak rising trend (Figure 7F, LP window), with the slope of a linear regression at 0.55, which was not significantly different from 0 since the 99% confidence interval of the slope was [−0.23 1.34].

To compare with those results using the LP data window, we repeated the analysis for the three cue-on period windows (Figure 7F). For CO1, from 0.5 to 1.0 s after cue onset while directional movement was being performed, improvement of Z-scored classification accuracy was significant (slope: 0.98; 99% confidence interval of slope: [0.25 1.71]). For CO2, from 1.0 to 1.5 s after cue onset when directional movements were mostly completed, improvement of Z-scored classification accuracy was significant as well (slope: 1.48; 99% confidence interval of slope: [0.79 2.17]). An improvement of Z-scored classification accuracy (slope: 1.12; 99% confidence interval of slope: [0.40 1.84]) was also observed in CO3, from 1.5 to 2.0 s after cue onset which was right before extension of response levers. When the regression analysis was carried out on individual rats, regression slopes for cue-on period data windows were significantly greater than zero (*t*-test; CO1, *p* < 0.01; CO2, *p* < 0.005; CO3, *p* < 0.05), but not significantly different from zero for LP (*t*-test, *p* > 0.39). When classification accuracies were averaged for each of the three equal-length learning stages, Z-scored classification accuracy increased gradually over stages for each of the three cue-on period windows (CO1: −0.76, −0.37, and 1.13; CO2: −0.86, −0.24, and 1.10; CO3: −1.05, 0.11, and 0.94; ANOVA, 1st vs. 3rd stage, *p* < 0.05), but remained leveled during the last two stages for LP window (−1.15, 0.67, and 0.48; ANOVA, 2nd vs. 3rd stage, *p* > 0.9). Figure 7G illustrates the Z-scored classification accuracies in the three stages for the four data windows, and again consistent increment over the three stages was found in cue-on data windows but not in the LP window. Taken together, enhanced discriminability of neural activity patterns over the entire learning process was mainly found during the cue-on period, but not the LP period when rats actually pressed levers.

To further validate the results from SVM based decoding analyses presented above, we used linear discriminant analysis (LDA) as a second classification method. Similar results from LDA classifiers were obtained as those reported in Figures 7E–G.

**Figure 8:**
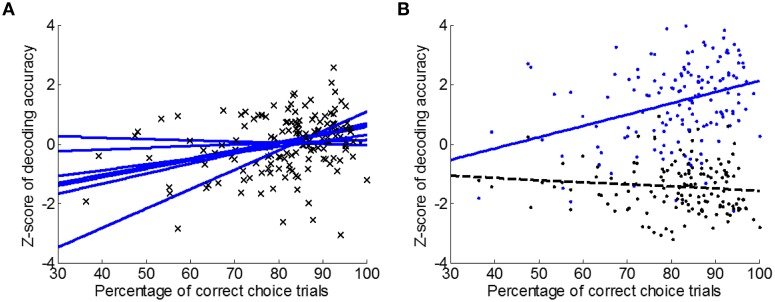
**SVM decoding performance vs. behavioral accuracy. (A)** Linear regression of Z-scored SVM decoding accuracy (CO data window, 500 ms bins) against the percentage of correct choice trials (i.e., L-L and R-R trials) for individual rats (*n* = 7). Increased decoding accuracy was observed in six out of seven rats. **(B)** Decoding accuracy using spatiotemporal patterns (blue; CO data window, 500 ms bins) improved as behavioral performance improved, but not so when ensemble mean firing rate over the entire CO data window (1500 ms bin) was used for decoding (black).

Regression analysis was also performed between classification accuracy (CO data window, 500 ms bins) and the percentage of trials with correct directional choice in single sessions. Results of the seven rats were plotted in Figure 8A individually. The linear regression slope was significantly greater than zero (*t*-test, *p* < 0.05). Thus, decoding of directional choice using neural activity did improve as rats made progress on the learning task.

To show that spatiotemporal patterns indeed facilitated the observed improvement in directional choice decoding, the mean firing rate of the neural ensemble over the whole CO data window (1500 ms bin, 1-D data samples) was used for classification as a comparison. Regression slope of classification accuracy against the percentage of correct choice trials (i.e., L-L and R-R trials) when using the 1-D data samples is −0.0071 (Figure 8B, black; *n* = 7), which is not significantly different from zero (99% confidence interval at [−0.0221 0.0078]). When spatiotemporal patterns (CO data window, 500 ms bins) were used for classification, the regression slope is significantly steeper (ANCOVA, *p* < 0.05), which is 0.0380 with 99% confidence interval at [0.0147 0.0612]. Therefore, neural adaptation associated with directional choice learning is better described by spatiotemporal activity patterns than a low resolution neural activity representation.

## Discussion

Seven out of nine rats successfully learned to perform a directional choice task from a naïve state. Using trial-and-error, they were able to associate a light cue with a same side lever pressing. Based on rat behavioral data, we observed that rat movement trajectory and the act of lever press became stereotyped within the first few days and therefore, the motor skill learning factor could be excluded from our analysis of associative learning. In this study, we focused on analyzing neural data from the seven rats during the cue-on period when they made directional choice decisions. Our results showed that the ensemble mean firing rate over all L-L and R-R trials appeared level over learning sessions. The ensemble mean rate difference between L-L and R-R trials did not show a strong trend of change as task learning progressed either (Figure 5). However, when using SVMs to decode directional choice from spatiotemporal neural activity patterns, there was a clear upward trend of SVM decoding accuracy over learning sessions. Correspondingly, there was a clear upward trend in discriminability of neural patterns between left side and right side choices (Figure 7). These findings suggest that neural adaptation in rat's motor cortical areas during learning of the directional choice task may lie in the spatiotemporal firing pattern of neural ensembles.

### Adaptation of spatiotemporal neural activity pattern during task learning

SVM classifiers were constructed to discriminate neural activity patterns associated with directional choices. During our analysis using SVM, care was given to ensure compatibility when comparing results over learning sessions. First, the same linear kernel SVM model was used for all analyses. Second, each and every classifier was trained with the same numbers of trial samples and tested with the same numbers of samples in the same session (Figures 6–8). Third, we reported classification accuracy in each session using an averaged result of 100 independent SVM classifiers with randomly selected training and test data samples. As such, SVM classification performance over sessions as reported in Figures 7, 8 should be characteristic of neural activity patterns as they adapted with learning.

In this study, we treated single units recorded from the same electrode in different sessions as independent unit records. Actually, our results reported in this study were based on neural ensembles which consisted of all units recorded simultaneously during one session from the same rat. Therefore, we did not intentionally identify and track same neurons over recording sessions. This approach was used before (Cohen and Nicolelis, 2004) and it is adopted in this study since all our results are based on ensemble neural activity.

Motor skill learning is a possible confound when analyzing neural activity in the cue-on period. However, we observed that rats became accustomed to directional movement and response lever press faster than the associative learning aspect of the task. Rat's directional movement became stereotyped quickly after the first few sessions (Figure 3D and Yuan et al., 2014) and the response latency of lever press decreased during the first three sessions (Figure 3B). Therefore, initial motor skill learning could not explain neural adaptation along an entire learning process lasted for weeks.

There may be other confounding factors in addition to motor skill learning during the first few learning sessions. Notice that right after a press on the center ready lever, at which time the directional cue was presented, there was a 2 s cue-on period. Rats usually moved from the center position to the location of their chosen response lever waiting for the extension of the response levers at the end of the cue-on period. Conceivably during this 2 s window, a rat's anticipation of lever extension and planning of lever press could possibly induce neural modulation. However, behavioral data showed that rats quickly became accustomed to performing the task routine after the first few days as indicated by their stereotyped movement and stable response latency. Therefore, they could quickly become habituated to the extension of response levers as well as the planning of the routine act of response lever press. From that, those potential confounding factors may be excluded from possible reasons for reported neural adaptation.

Would the reported neural pattern adaptation be explained by the repetition of directional movements? Our previous analysis showed that rat directional movement was mostly completed within CO1 window (Yuan et al., 2014), and there was no obvious or systematic movement during CO2 and CO3 windows (Figure 1B). Improved discriminability of neural patterns, however, was observed not only in CO1 but also in CO2 and CO3 windows (Figure 7F). Response lever press was another action repeatedly performed by the rats during learning of the task. However, we did not observe significantly improved discrimination of neural patterns in the LP data window (Figure 7F). As discussed above, these actions quickly became stereotypical and turned into learned motor skills. Previous primate studies showed that mere repetition of familiar actions did not induce systematic changes in motor cortical neural firing activity (Paz and Vaadia, 2004; Rokni et al., 2007). Taken together, the observed firing pattern adaptation during cue-on period was unlikely to be associated with repeated performance of task related actions.

Reward based stimulus-response association learning is another aspect that may affect neural modulation in the rat frontal areas that we recorded from. Previous studies showed that reward-related action selection is believed to be mediated by the corticostriatal circuitry, linking prefrontal (PFC), premotor, sensorimotor cortices, and the striatum (Balleine et al., 2007). As two important nodes within this circuit of primates, Pasupathy and Miller (2005) found rapid changes in striatum but slow adaptation in PFC during an associative learning task, where the time course of PFC activity had a significantly stronger correlation with the gradual improvement in task performance. In accordance with their findings, the adaptation we observed in rat's frontal areas also correlated with slow improvement of behavioral performance. Given the above considerations, the observed neural adaptation during the cue-on period could be attributed to learning the correct stimulus-response association.

In the rat brain, both AGm and AGl project to basal ganglia (Reep et al., 1987; Cheatwood et al., 2003; Alloway et al., 2009), and both areas receive inputs from basal ganglia through the thalamus (Donoghue and Parham, 1983; Reep et al., 1984). Rat AGm and AGl have connections with a variety of frontal cortical areas as well (Reep et al., 1984, 1990; Hoover and Vertes, 2007). Therefore, these rat motor cortical areas could be in the loop of the reward-related decision making circuit. The neural pattern adaptation in motor cortical ensembles reported here provides neurophysiological evidence for a role of rat motor cortical areas in learning stimulus-response associations, which could be mediated by this neural circuit when rewarded directional choices were learned.

### Ensemble mean rate vs. spatiotemporal activity pattern

Despite the well-observed phenomenon of dynamic neural modulation in single neurons, relatively uniform firing rates in cortical ensembles have been reported in different brain areas of both primates and rodents while animals performed different tasks (Hoffman and McNaughton, 2002; Carmena et al., 2003; Costa et al., 2006; Pantoja et al., 2007). But that does not include studies that involve learning tasks except a few. In an associative learning task, Cohen and Nicolelis (2004) reported unchanged rate difference over the recorded neural ensemble during the early days, specifically day 2 and day 3. When monkeys were performing a stimulus-response association learning task, firing rate change in single units of motor cortical areas was reported (Mitz et al., 1991; Chen and Wise, 1995; Brasted and Wise, 2004), but it was less certain if and how firing rate of a neural ensemble would change over learning. Here, we report a relatively stable ensemble mean firing rate and ensemble mean rate difference between left and right side choices over a period of about 20–30 sessions covering the entire time course of associative learning. Our results may suggest a balanced increase and decrease in single unit firing rates, which may have contributed to a stable motor cortical ensemble mean firing rate during associative learning. This result is supportive of “the conservation of firing” principle proposed by Nicolelis and Lebedev (2009).

To gain understanding of neural coding beyond ensemble averaged firing rates, investigations of spatiotemporal activity patterns at a fine resolution have brought up new insight on fundamental neural mechanisms in visual attention (Heinze et al., 1994), odor representation (Laurent et al., 1996; Spors and Grinvald, 2002; Rennaker et al., 2007), auditory processing (Kayser et al., 2009), vibrissa deflection coding (Petersen and Diamond, 2000), contextual encoding (Hyman et al., 2012), sequence learning (Ma et al., 2014), and rule learning (Durstewitz et al., 2010), to name a few. In a reaction time study (Laubach et al., 2000) using a rat model, the overall firing rates of the AGm and AGl ensembles did not change significantly but prediction of trial outcome of either correct or error based on spatiotemporal activity patterns improved over learning sessions. Our results appear along similar lines. However, the two experimental protocols are different in a few aspects. The Laubach et al. (2000) experiment used a single stimulus (vibrotactile or auditory) and a lever release for rats to report their detection of presence of sensory cue. In our experiment, alternative choices were associated with distinct stimuli. Besides, Laubach et al. (2000) compared lever release either instructed by a stimulus (correct) or executed spontaneously without stimulus presence (error), while we compared instructed left side and right side choices (both were correct) under distinct cues. This may help rule out confounding factors such as the occurrence of sensory stimuli and prediction of rewards (Carandini and Churchland, 2013).

In the Cohen and Nicolelis (2004) study, prediction of left and right side movements by M1 neural ensembles improved from the first day to the next 2 days. Unfortunately, results in the remaining 8 days as rats' performance continued to improve until reaching a plateau were not available. Here we monitored neural activity patterns through the entire process of associative learning, and demonstrated improved discriminability of spatiotemporal firing patterns in motor cortical ensembles.

### Rat motor cortical areas and associative learning

The rat AGl area has been considered to correspond with primate primary motor (M1) cortex (Donoghue and Wise, 1982; Donoghue and Parham, 1983). On the other hand, the rat AGm area refers to the medial subdivision of the agranular field of the frontal cortex which differs from the lateral subdivision (AGl) on cytoarchitectonic grounds (Donoghue and Wise, 1982). Other terms referring to this area used in literature include medial precentral area (PrCm, Krettek and Price, 1977), frontal cortical area 2 (Fr2, Zilles, 1985), and secondary motor area (M2, Paxinos and Watson, 2005; MOs, Swanson, 1998). Leaving the inconsistent nomenclature aside, rat AGm was proposed to be homologous to premotor cortex, supplementary motor area, and frontal and supplementary eye fields in primates (Donoghue and Wise, 1982; Reep et al., 1987; Van Eden et al., 1992; Condé et al., 1995; Sul et al., 2011). However, a clear rat AGm homology in primates has yet to be proved convincingly. Nonetheless, previous neuropsychological studies showed that lesions of rat AGm impaired both the retrieval (Passingham et al., 1988) and the acquisition (Winocur and Eskes, 1998) of visuomotor conditioning, which suggest a role for AGm in stimulus-response associative learning. In line with these reports, we observed firing pattern adaptation during associative learning within the motor cortical neural ensembles, which consisted of AGm neurons and AGl neurons.

It is worth mentioning that in most of the primate studies on associative learning, the animals usually had acquired certain stimulus-response association through long and extensive training. And then, animals would learn a novel pairing (Chen and Wise, 1995; Brasted and Wise, 2004) and/or the reversed pairing (Pasupathy and Miller, 2005; Histed et al., 2009) during one complete recording session. Sometimes the animals would learn a variant of the trained sensorimotor tasks (Mitz et al., 1991; Li et al., 2001; Genovesio et al., 2014), which also could be completed within one session. In these cases, the complete time course of learning could take place in tens of minutes or a single recording session. This may be too soon to result in long-lasting synaptic changes, as suggested by Histed et al. (2009). In our experiment, it took rats several weeks to master the directional choice task. This may allow substantial neural adaptation to take place, possibly through changes at the synaptic level.

Synaptic plasticity has long been hypothesized for being an important neurochemical foundation of learning and memory (Malenka and Bear, 2004; Gilson et al., 2010), and its necessity has been well-supported (Martin et al., 2000). Rat motor cortex is highly capable of functional and structural changes even in adulthood. Reorganization of motor maps has been observed in various experiments (Sanes et al., 1990, 1992; Lee et al., 2003), including animals learning a motor skill (Nudo et al., 1996; Kleim et al., 1998, 2004). Cortical synaptogenesis has been reported during motor training (Jones et al., 1999; Kleim et al., 2004). And recent studies demonstrated learning-induced dendritic spine changes in rodents performing motor tasks (Xu et al., 2009; Yang et al., 2009; Wang et al., 2011). While these changes were related to learning of certain motor skill, whether learning stimulus-response association would induce such changes in rat motor areas is unclear. Given the lengthiness of the task, rats in our experiment may have a chance to experience synaptic modification during the learning process that lasted several weeks. Consequently, an enhanced spatiotemporal neural representation may become increasingly predictable of directional choice.

### Conflict of interest statement

The authors declare that the research was conducted in the absence of any commercial or financial relationships that could be construed as a potential conflict of interest.
